# Detecting Recombination Hotspots from Patterns of Linkage Disequilibrium

**DOI:** 10.1534/g3.116.029587

**Published:** 2016-05-24

**Authors:** Jeffrey D. Wall, Laurie S. Stevison

**Affiliations:** * Institute for Human Genetics, University of California, San Francisco, California 94143; † Department of Biological Sciences, Auburn University, Alabama 36849

**Keywords:** recombination hotspots, linkage disequilibrium, composite likelihood

## Abstract

With recent advances in DNA sequencing technologies, it has become increasingly easy to use whole-genome sequencing of unrelated individuals to assay patterns of linkage disequilibrium (LD) across the genome. One type of analysis that is commonly performed is to estimate local recombination rates and identify recombination hotspots from patterns of LD. One method for detecting recombination hotspots, LDhot, has been used in a handful of species to further our understanding of the basic biology of recombination. For the most part, the effectiveness of this method (*e.g.*, power and false positive rate) is unknown. In this study, we run extensive simulations to compare the effectiveness of three different implementations of LDhot. We find large differences in the power and false positive rates of these different approaches, as well as a strong sensitivity to the window size used (with smaller window sizes leading to more accurate estimation of hotspot locations). We also compared our LDhot simulation results with comparable simulation results obtained from a Bayesian maximum-likelihood approach for identifying hotspots. Surprisingly, we found that the latter computationally intensive approach had substantially lower power over the parameter values considered in our simulations.

Homologous recombination is a fundamental biological process. In most organisms, it is necessary for the proper alignment and segregation of chromosomes during meiosis, influences the efficacy of natural selection, and is the primary determinant of the strength of allelic associations (*i.e.*, linkage disequilibrium, or LD) across the genome. It is now well established that recombination rates vary tremendously across the genome, and that many species have recombination ‘hotspots’, narrow regions (<2 kb) where the recombination rate is much higher than the rate in the surrounding sequence (Petes 2001; Buard and de Massy 2007). In humans, for example, sperm typing studies have experimentally identified dozens of hotspots (*e.g.*, Jeffreys *et al.* 2001, 2005; Sarbajna *et al.* 2012), and pedigree-based studies have identified broad-scale variation in recombination rates (Broman *et al.* 1998; Kong *et al.* 2002, 2010). In most eukaryotic species direct estimates of recombination are time-consuming, and not easily scaled up to studies of fine-scale recombination rate variation across the whole genome (but see Mancera *et al.* 2008; Comeron *et al.* 2012).

An appealing alternative is to estimate local changes in recombination rate using patterns of LD at single nucleotide polymorphisms (SNPs) (Crawford *et al.* 2004; McVean *et al.* 2004; Chan *et al.* 2012). These methods have been used to construct fine-scale recombination maps in humans (Myers *et al.* 2005; 1000 Genomes Project Consortium 2010), great apes (Auton *et al.* 2012; Stevison *et al.* 2016), *Drosophila melanogaster* (Chan *et al.* 2012), *Arabidopsis thaliana* (Horton *et al.* 2012), *Medicago truncatula* (Paape *et al.* 2012), house mouse (Brunschwig *et al.* 2012), and dogs (Axelsson *et al.* 2012; Auton *et al.* 2013). While these recombination maps can suggest the locations of many potential recombination hotspots, separate methodology (*e.g.*, a hypothesis test) is needed to statistically test whether any particular region is actually a hotspot. Otherwise, a local increase in estimated rate could reflect chance variation in the genealogical process, rather than point to a real hotspot. While several methods for statistically identifying hotspots have been proposed (Crawford *et al.* 2004; McVean *et al.* 2004; Fearnhead 2006; Li *et al.* 2006; Wang and Rannala 2009), almost all are computationally expensive enough to limit their use to candidate regions (Crawford *et al.* 2004; Tsai *et al.* 2010; Chan *et al.* 2012) or to sparse genotype data (Axelsson *et al.* 2012). In most whole-genome applications to date, one method, LDhot (McVean *et al.* 2004), has been used to identify recombination hotspots in humans, chimpanzees, dogs, and *A. thaliana*. The accuracy of this method for identifying true recombination hotspots though is unknown.

The use of population genetic methods to computationally predict hotspot locations has led to several major insights into the evolution of fine-scale recombination rates. Notably, computational analyses identified a degenerate 13 bp motif (CCNCCNTNNCCNC) that is overrepresented in predicted human hotspots relative to matched coldspots (Myers *et al.* 2008), and subsequent work has shown that this motif matches the predicted binding domain for the PRDM9 gene (Baudat *et al.* 2010; Myers *et al.* 2010; Brick *et al.* 2012). PRDM9 trimethylates lysine 4 of histone H3 (H3K4me3) (Hayashi *et al.* 2005), and H3K4me3 marks are associated with double strand breaks and recombination in both yeast and mice (Borde *et al.* 2009; Buard *et al.* 2009). Variation in PRDM9 is associated with differences in fine-scale recombination rates and hotspot usage across the genome (Baudat *et al.* 2010). Thus, the use of these computational methods has identified a new and key player in mammalian recombination. In addition, a comparison of fine-scale recombination maps in humans and chimpanzees found that chimpanzees have recombination hotspots as well, but at different locations than human hotspots (Auton *et al.* 2012; but see Wang and Rannala 2014). This confirmed the results of previous analyses of much smaller data sets (Wall *et al.* 2003; Ptak *et al.* 2004; Winckler *et al.* 2005) and is in line with the theoretical expectations of biased gene conversion (Boulton *et al.* 1997; Coop and Myers 2007). PRDM9 evolves rapidly in metazoans (Oliver *et al.* 2009; Schwartz *et al.* 2014), and the different predicted PRDM9 binding motifs for humans and chimpanzees may explain why there is no (or little) overlap in hotspots between the two species. Thus, the discovery of this gene may also explain why hotspots have evolved so rapidly in apes.

The initial computational analyses failed to identify any particular sequence motif that is associated with predicted chimpanzee hotspots, which was ascribed to the high allelic diversity at PRDM9 in chimpanzees and the complex relationship between PRDM9 sequence and targeted binding sites (Auton *et al.* 2012; Billings *et al.* 2013). Moreover, though PRDM9 appears to be absent in plants and canids (Oliver *et al.* 2009), studies of SNP data in *A. thaliana*, *M. truncatula*, and *Canis lupus familiaris* found strong evidence for thousands of recombination hotspots in these three species (Axelsson *et al.* 2012; Horton *et al.* 2012; Paape *et al.* 2012; Auton *et al.* 2013). However, a similar study of recombination rate variation in flies identified fewer than 10 hotspots in different populations of *D. melanogaster* (Chan *et al.* 2012). Thus, computational methods have further highlighted intriguing similarities and differences among taxa.

There have been at least three separate implementations of LDhot (Myers *et al.* 2005; Auton *et al.* 2012, 2014), each with different criteria for calling hotspots. Until recently (Auton *et al.* 2014) no version of the program was publicly available. This has hampered our understanding of the extent to which published results reflect the true biological reality *vs.* limitations of the hotspot calling methodology. In this study, we implement our own version of LDhot (available from github at https://github.com/jdwall02/mlehot), and test the power of LDhot to detect true recombination hotspots over a range of model parameters appropriate for large mammals such as humans and great apes. We find that some previous implementations of LDhot have extremely low power, which might explain the results of Johnston and Cutler (2012). If many true hotspots have gone undetected, then an actual correlation between hotspot sequences and a sequence motif may have been missed, and the degree of sharing of hotspot locations across species (*e.g.*, Auton *et al.* 2012) may have been underestimated. We explore this possibility in greater detail below. We also compare the power and false positive rate of LDhot with a maximum-likelihood approach for estimating recombination rates and calling recombination hotspots (Wang and Rannala 2009).

## Materials and Methods

LDhot uses a composite likelihood framework based on the work of Hudson (2001) and McVean *et al.* (2002) and similar to the approach of HotspotFisher (Li *et al.* 2006). The Auton *et al.* (2012) implementation tests every 2 kb region (with a 1 kb increment) as a potential hotspot by analyzing the 200 kb region centered around the region of interest. Suppose the SNPs in the 200 kb region are S = {s_1_... s_n_}. A (composite) LRT statistic is calculated as

$$\text{R}=\frac{\underset{{\text{ρ}}_{\text{0}}{\text{,ρ}}_{1}}{\sup}\;\prod_{i=1}^{n-1}\prod_{j=i+1}^{n}\;\text{lik}\;{\text{(s}}_{\text{i}}{\text{,s}}_{\text{j}}{\text{|ρ}}_{\text{0}}{\text{,ρ}}_{1})}{\underset{\text{ρ}}{\sup}\;\prod_{i=1}^{n-1}\prod_{j=i+1}^{n}\;\text{lik}\;{\text{(s}}_{\text{i}}{\text{,s}}_{\text{j}}\text{|ρ})}$$ (1)

where lik (s_i_, s_j_ | ρ) is the two-site likelihood described before (Hudson 2001; McVean *et al.* 2002), ρ_0_ is the background recombination rate, and ρ_1_ is the recombination rate in the central 2 kb region. Critical values for R are estimated from null simulations that assume a constant recombination rate across the region (*i.e.*, ρ_0_ = ρ_1_). Auton *et al.* (2012) used ‘fixed S’ methodology for these simulations (Hudson 1993; Wall and Hudson 2001), with SNP locations fixed to be where SNPs appear in the actual data and ρ chosen to be equal to its estimated value (from LDhat). They tested each possible 2 kb region and identified those where the estimated *P*-value for R was <0.01. Then, overlapping regions were merged to form a list of candidate hotspot regions. These regions were filtered to reduce the false positive rate by eliminating ones >5 kb in size or with peak ρ estimate <5 /kb (estimated using LDhat).

We also studied two other approaches for calling hotspots using LDhot. Auton *et al.* (2014) used a smaller window size (100 kb) but the same basic approach for identifying candidate regions. Instead of a size or peak ρ estimate filter though, they required each hotspot region to contain at least one 2 kb window where the estimated *P*-value for R was <0.001. Our new approach here is to generate the same list of candidate regions as Auton *et al.* (2012), partition each region into nonoverlapping 1 kb windows, and keep only those windows for which the average ρ estimate (using LDhat) is ≥5/kb. A brief summary of the differences between LDhot implementations is summarized in Table 1.

**Table 1:** Key differences between three implementations of LDhot

Method	Auton *et al.* 2012	Auton *et al.* 2014	mlehot
Window size	200 kb	100 kb	20 kb
*P*-value cutoff	0.01	0.001	0.01
Max. size	5 kb	None	None
LDhat	Peak must be ≥5/kb	–	Intersection with 1 kb regions with ρ ≥ 5
Simulations	Region-specific	Region-specific	Lookup table

For all three hotspot calling protocols, we found that the publicly available version of LDhot (Auton *et al.* 2014) was too slow for running power calculations, since the null distribution for R is estimated separately for each test window. We implemented our own version, similar to the method of Myers *et al.* (2005) – we run null simulations in advance, store the results in a large lookup table, and use these to repeatedly estimate the significance values for observed values of R across the genome. Specifically, for a window size of X kb we run coalescent simulations (*cf*. Hudson 2002) of X kb regions across a broad range of mutation and recombination rates (assuming the recombination rate is constant per base pair). For each simulation, we tabulated R, the number of segregating sites S, and the estimate of ρ (*cf*. Hudson 2001). Then, when analyzing an actual X kb region, we calculate S and ρ, then use simulations with S and ρ near the actual values for determining the null distribution for R. So, while the Auton *et al.* (2012) approach uses ‘fixed S’ simulations and parametric bootstrapping (for ρ), we use standard coalescent simulations and condition (in the standard statistical sense) on the observed value of S and the observed estimate of ρ (with an implicitly flat prior for θ and ρ). Our simulations took X = 20, 50, 100, or 200 kb, and we ran ∼5 × 10^6^ simulations for each value of X. Source code and executables for running all three implementations of LDhot are available at http://github.com/jdwall02/mlehot

To estimate the power of the different hotspot calling protocols, we assumed a sample size of n = 30 haploid sequences, a scaled mutation rate of θ = 1/kb, and a scaled background recombination rate of ρ = 0.5/kb. We simulated 100 different 1 Mb regions, each containing eight different 2 kb hotspots with scaled recombination rates of 5, 10, 25, or 50/kb. We defined the power as the proportion of actual hotspot sequence that was identified as a hotspot using LDhot. Similarly, the false positive rate was calculated as the proportion of actual nonhotspot sequence that was called as a hotspot using LDhot, and the false discovery rate was defined as the proportion of called hotspot sequence that was not actually contained in a recombination hotspot. (Note that these definitions differ from those of some previous studies.) Additional simulations considered a wider range of sample sizes (*n* = 16–42 haploid sequences) or other scaled background recombination rates (ρ = 0.1, 0.2, 1, or 2.5 per kb). These latter simulations had actual hotspot recombination rates that were 10, 20, 50, or 100 times the background rate and a new hotspot calling criteria of ρ ≥ 10 times the background rate (estimated from LDhat). One final set of simulations had *n* = 30, a scaled mutation rate of θ = 5/kb, and a scaled background recombination rate of ρ = 0.5–5/kb.

We also used Inferrho, a Bayesian full-likelihood method for calling recombination hotspots developed by Wang and Rannala (2009). We used both the current version of the program (IRv1) as well as the originally published version (INFERrho, obtained from Y. Wang). Inferrho estimates a posterior probability that any particular genomic location is contained in a recombination hotspot. We used the same hotspot calling criteria as Wang and Rannala (*i.e.*, HT_1_ = 5, HT_2_ = 2.5) on 20 simulated 1 Mb regions with recombination hotspots (background ρ = 0.5/kb; other parameters are described in the previous paragraph). For computational tractability, we broke each simulated region into overlapping 7 kb subregions (each overlap being 2 kb), and analyzed each subregion separately after trimming off 1 kb from each end. We also only analyzed a subset of the nonhotspot regions to estimate the false positive and false discovery rates. Finally, we reanalyzed the original 100 simulations described in Table 1 of Wang and Rannala (2009), using their original implementation, simulation parameters (burnin = 10^4^ steps, MCMC chain = 10^5^ steps), and criteria for calling hotspots. We also reran half of these simulations with a burnin of 2 × 10^5^ steps and an MCMC chain of 10^6^ steps and found our results to be unchanged (results not shown).

### Data availability

The authors state that all data necessary for confirming the conclusions presented in the article are represented fully within the article.

## Results

To assess the power and false positive rate of LDhot, we implemented our own version that is fast enough to allow for power calculations on simulated data. We then estimated LDhot’s power under a range of window sizes and recombination hotspot intensities, using the same basic protocol as Auton *et al.* (2012). Unlike previous simulation studies (Wang and Rannala 2009; Auton *et al.* 2014), ours uses a background recombination rate that is more appropriate for human or great ape data.

**Figure 1:**
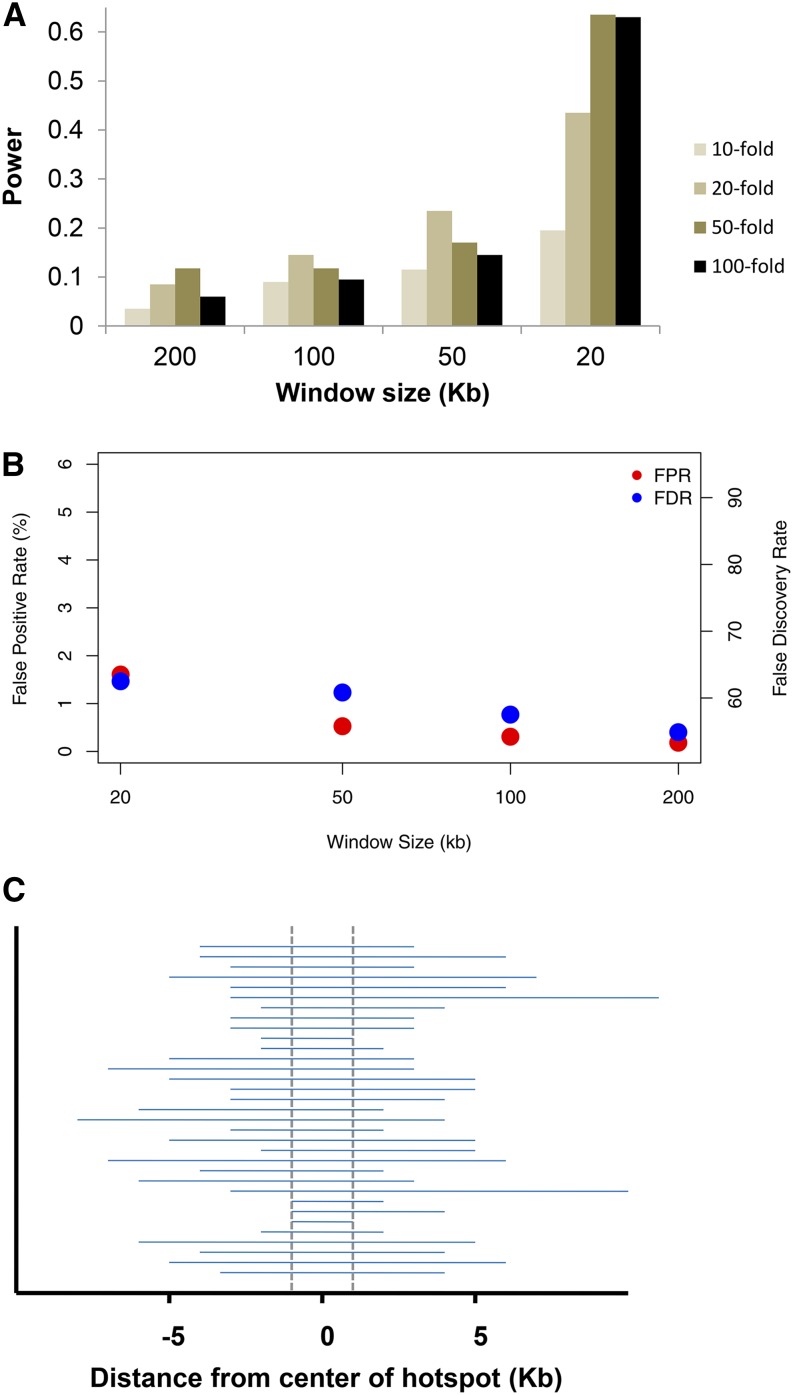
(A) Power to detect a hotspot as a function of the window size (x-axis) and the strength of the hotspot using the protocol of Auton *et al.* (2012). Background ρ is 0.5/kb. See text for further details. (B) False positive rate for estimated hotspots, defined as the proportion of estimated hotspot sequence that was not actually a simulated hotspot. (C) Estimated hotspot locations without the length limitation for a 100 kb window and a 50-fold hotspot. Dashed vertical lines show location of actual hotspot. Results are similar for other parameter combinations.

The power results are shown in Figure 1A while the false positive rates and false discovery rates (FDR) are shown in Figure 1B. Two trends are easily apparent. First, the power increases as the window size decreases, with an ∼fivefold increase in power for a 20 kb window size compared with a 200 kb window size. Second, stronger hotspots are not always easier to detect, and the power to detect recombination hotspots under any scenario is quite modest, topping out at 63% for a 20 kb window size and a 50-fold increase in the recombination rate and with a low of 3% power for a 200 kb window size and a 10-fold increase in the recombination rate. The FDR is relatively high at 55–63%. This, plus the lower power, suggests that existing methods might have substantial room for improvement.

The general trends described above are a consequence of the composite likelihood approach used by LDhot, and the protocol used to manage the false positive rate. Since all pairs of sites within a window are used in the likelihood calculations, most of the pairs in large (*e.g.*, ≥100 kb) windows will be uninformative about the precise location of a hotspot. So, if a hotspot is called for a region, it tends to be large (*i.e.*, >5 kb in length), and these large regions are subsequently excluded. Similarly, when the recombination hotspot is strong (50-fold or 100-fold increase), LDhot has more trouble with hotspot localization, leading to large hotspot regions that are also excluded. To illustrate this, Figure 1C shows the estimated hotspot locations relative to the true hotspot location for a 100 kb window size and a 50-fold hotspot. While 85% of the simulated hotspots were identified using a *P* < 0.01 cutoff, the vast majority of these were excluded from Figure 1A due to their size. This leads to decreasing power for increasing window size.

**Figure 2:**
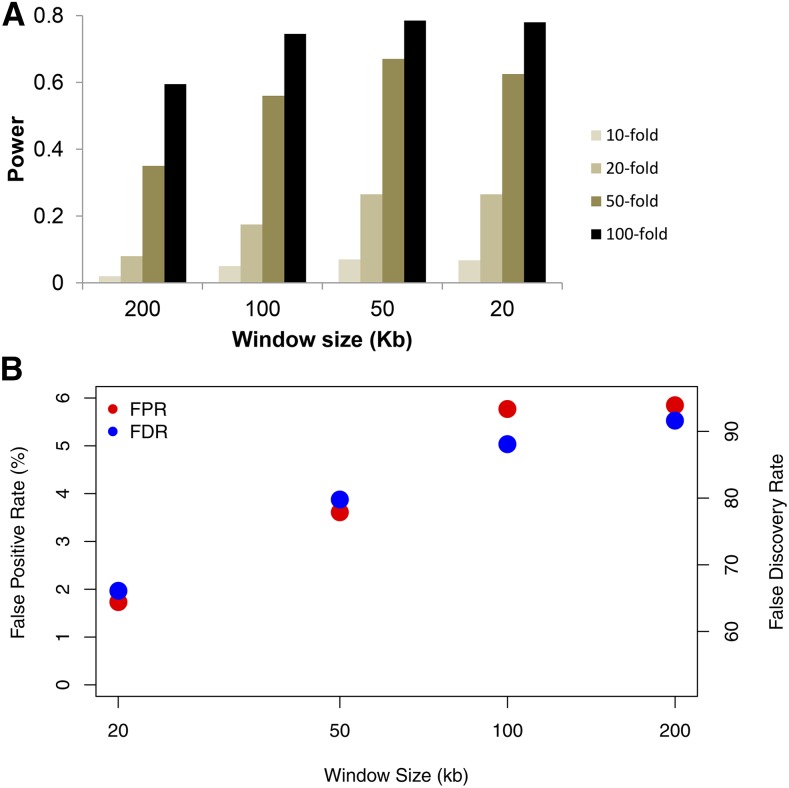
(A) Power to detect a hotspot as a function of the window size and strength of the hotspot using the protocol of Auton *et al.* (2014). Results are directly comparable to those of Figure 1A and Figure 3A. (B) False positive rate for estimated hotspots, defined as the proportion of estimated hotspot sequence that was not actually a simulated hotspot.

If we drop the 5 kb length restriction, then the power (and false positive rate) increase substantially. Figure 2 shows the power and false positive rates if we use the Auton *et al.* (2014) criteria for calling hotspots instead. This approach adopts a stricter *P* < 0.001 cutoff for identifying a candidate region as a hotspot, but does not have any length restriction on the size of the region identified (see *Materials and Methods* for a more precise description). While the power increases substantially when comparing Figure 1A to Figure 2A, this is achieved at a cost of having a false positive rate that ranges from 1.7 to 5.8% and a FDR of 66–92% (Figure 2B). For these simulations, the average size of the identified hotspot regions varies from 5.1 kb (20 kb window size) to 23.3 kb (200 kb window size). Clearly, a 20 kb “hotspot region” is not very informative, even if it does contain a true recombination hotspot.

**Figure 3:**
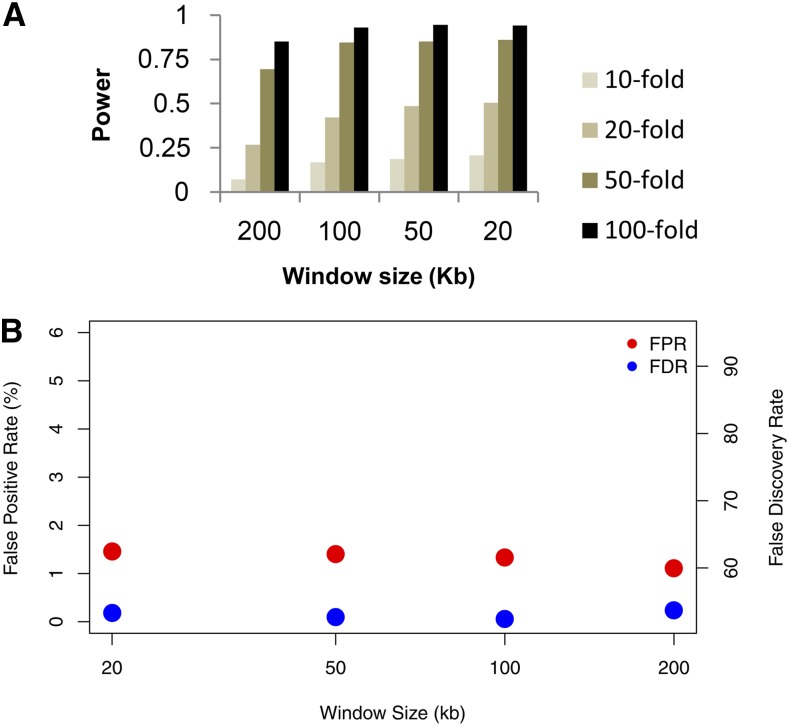
(A) Power to detect a hotspot as a function of the window size and strength of the hotspot using our new protocol. Results are directly comparable to those of Figure 1A and Figure 2A. (B) False positive rate for estimated hotspots, defined as the proportion of estimated hotspot sequence that was not actually a simulated hotspot.

We also tried another approach for hotspot calling, by analyzing each 1 kb region separately, and requiring both *P* < 0.01 (using LDhot) and ρ ≥ 5/kb (using LDhat) for a region to be called a hotspot. Under this protocol, the power to detect hotspots is much higher than for the previously proposed methods (Figure 3A). For a 20 kb window size, the power ranges from 21% for a 10-fold hotspot to 94% for a 100-fold hotspot, compared with 17% and 40% for the Auton *et al.* (2012) approach and 7% and 78% for the Auton *et al.* (2014) approach. The false positive rate (Figure 3B) is intermediate between the results of Figure 1B and Figure 2B, while the FDR ranges from 52 to 54%, lower than for the previous methods used for calling recombination hotspots. We conclude that our new method for calling hotspots using LDhot is better than the previous approaches, and that smaller window sizes (*e.g.*, 20 kb) should be used when analyzing dense SNP or resequencing data.

**Figure 4:**
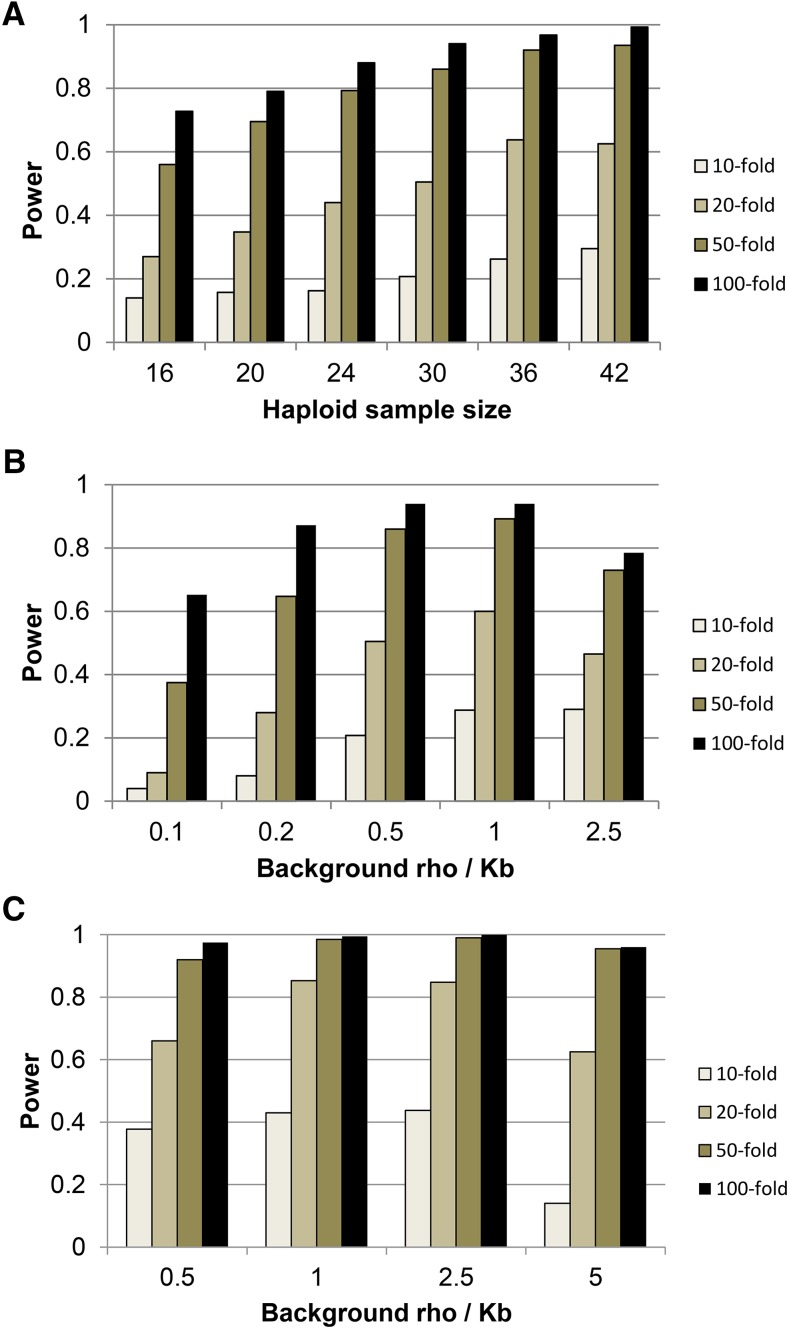
Power to detect hotspots using our new protocol over (A) different haploid sample sizes, (B) different background recombination rates, and (C) higher mutation and (background) recombination rates.

To explore how sensitive these results are to the particular parameter values used, we also estimated the power to detect recombination hotspots for a range of haploid sample sizes and different background recombination rates. In almost all simulations, our new hotspot calling protocol has substantially higher power and lower false discovery rate than the previously described protocols (Auton *et al.* 2012, 2014), even when we use the same window size for each. For brevity, we include only the results of the new hotspot calling protocol on 20 kb windows. As expected, power increases with increasing sample size (Figure 4A), with some leveling off once *n* > 30. Additionally, we find that hotspots are easier to detect when the background recombination rate is intermediate (ρ = 0.2–1/kb, *cf*. Figure 4B), presumably because for low background rates the levels of LD are high even in recombination hotspots and for high background rates the levels of LD are somewhat low even for background regions. Additional simulations suggest that power is also increased (and FDR decreased) when the baseline levels of genetic variation (*e.g.*, θ = 4 Nμ) are higher. Figure 4C shows simulation results with a fivefold higher amount of polymorphism (θ = 5/kb). The increased information content with the higher density of SNPs leads to the increase in power, though this breaks down at the highest recombination rates (presumably due to the low levels of LD even in nonhotspot regions). These results suggest that the ease with which recombination hotspots can be identified in a given species from patterns of LD depends strongly on the values of fundamental biological parameters such as the effective population size, mutation rate, and recombination rate.

Finally, to see whether the composite likelihood approach of LDhot reduces power compared with computationally intensive full-likelihood approaches, we compared a subset of the results shown in Figure 3 with comparable results obtained from Inferrho (Wang and Rannala 2009). The results, shown in Table 2, are striking. Compared with LDhot, Inferrho has much smaller power, false positive rate, and false discovery rate. Inferrho essentially never finds weak hotspots (*e.g.*, 10- or 20-fold increase in recombination rate) and is very conservative even for the strongest simulated hotspots. On the other hand, when Inferrho does call a region a hotspot, there is a very high likelihood that it really is one (FDR ∼4%). If we use a more liberal definition of power, where any overlap between a called hotspot and an actual hotspot is counted as a hit, Inferrho has moderate power ranging from 2.5% for a 10-fold hotspot to 62.5% for a 100-fold hotspot.

**Table 2:** Comparison between our implementation of LDhot and Inferrho over 20 different 1 Mb regions

Power (%)	LDhot	Inferrho
10-fold hotspot	17.5	0.9
20-fold hotspot	48.8	2.8
50-fold hotspot	87.5	15.0
100-fold hotspot	95.0	26.5
False positive rate (%)	1.59	<0.2
False discovery rate (%)	55.6	≥4.1

Due to computational limitations, we could not calculate exact values of the false positive and false discovery rates for Inferrho.

## Discussion

Our simulation results suggest that the efficacy of LDhot in detecting recombination hotspots is quite sensitive to the particular implementation used in the analyses. Specifically, the larger window sizes used in previous studies (*e.g.*, 200 kb in Auton *et al.* 2012) lead to greatly reduced power and a higher false positive rate when compared to smaller window sizes (*e.g.*, 20 kb in Figure 1, Figure 2, and Figure 3). Qualitatively, this is because of the nature of the underlying composite likelihood used by LDhot – all pairs of sites are used within a window, including ones that are uninformative due to their distance from the central test region. LDhot’s original formulation (McVean *et al.* 2004) was optimized for human SNP data where the density of markers was relatively low. Now that full resequencing data are available, much smaller window sizes are needed for accurately estimating background *vs.* putative hotspot recombination rates. While our simulations are not exhaustive, they suggest that the optimal setup is to have the smallest window size that can accurately estimate background recombination rates. For species with human-like evolutionary parameters, this involves a window size of 20 kb (or perhaps slightly smaller). For species with a much higher level of diversity, a 10 kb or smaller window size would be appropriate (results not shown).

Our simulations also found that even for a fixed window size, the protocols used for identifying recombination hotspots have a strong influence on LDhot’s power and false positive rate. While the new protocol proposed here seems superior to previous implementations (Auton *et al.* 2012, 2014), we caution that our approach is *ad hoc* and that even better protocols are likely available. We also note that using a suboptimal version of LDhot can have real consequences when analyzing actual data. Auton and colleagues found no evidence of an association between predicted PRDM9 binding sites and recombination hotspots in chimpanzees (Auton *et al.* 2012). The low power of their LDhot implementation led to fewer called hotspots (<5000), which in turn limited their power to detect any association between sequence motifs and hotspots. Our analysis with an improved LDhot identified twice as many hotspots, and found sequence motifs (corresponding to predicted PRDM9 binding sites) that are overrepresented in recombination hotspots across several great ape species, including chimpanzees, bonobos, and gorillas (Stevison *et al.* 2016).

Finally, we found that even though LDhot has some serious drawbacks from a statistical standpoint, the computationally intensive full-likelihood approach of Wang and Rannala (2009) has much lower power. These results were unexpected, in part because of the much higher power (74–92%) reported by the authors (Table 1, Wang and Rannala 2009). While part of the difference can be ascribed to the particular parameter values used (*e.g.*, we used a background recombination rate of ρ = 0.5/kb while they used ρ = 0.06/kb), the performance of Inferrho was still surprising. To examine this further, we obtained the 100 simulated data sets analyzed by the authors (with hotspot ρ = 10/kb) and reanalyzed them using both the currently distributed version of Inferrho (IRv1) and the original version (INFERrho) used in Wang and Rannala (2009). Using the same simulation parameters and hotspot calling criteria as their original paper, we were not able to recreate their results (Table 3). These results were unchanged even when we increased the burnin and MCMC chain length by an order of magnitude (see *Materials and Methods*; results not shown). Consistent with the qualitative results of our previous simulations, we found both versions of Inferrho to have lower power and lower false positive rate than what was originally reported.

**Table 3:** Comparison between the results published in Wang and Rannala (2009, Table 1) and our computations of Inferrho using the same data sets

	Wang and Rannal (2009)*^a^*	IRv1	INFERrho
Power (%)	–*^a^*	19.0	28.3
False positive (%)*^b^*	4	0	0
Overlap (%)*^c^*	74	33	39

False positive refers to the Wang and Rannala (2009) definition – called hotspots that do not overlap at all with true hotspots.

a Cannot be determined from the original study.

b This refers to the Wang and Rannala (2009) definition – called hotspots that do not overlap with all true hotspots.

c This is Wang and Rannala’s (2009) definition of power – the proportion of true hotspots that overlap by at least 1 bp with a called hotspot.

We conclude that methods for identifying recombination hotspots should be tested thoroughly on simulated data and compared with each other across a wide range of parameter values to assess the efficacy of each and to determine which ones are the best to use. We hope that this study can be one step toward this goal.
